# Household treatment cost of breast cancer and cost coping strategies from a tertiary facility in Ghana

**DOI:** 10.1371/journal.pgph.0000268

**Published:** 2022-03-09

**Authors:** Kekeli Kodjo Adanu, Eyram Cyril Bansah, David Adedia, Moses Aikins

**Affiliations:** 1 Department of Surgery, School of Medicine, University of Health and Allied Sciences, Ho, Ghana; 2 Richard Novati Catholic Hospital, Sogakope, Ghana; 3 Department of Basic and Biomedical Sciences, School of Basic and Biomedical Sciences, University of Health and Allied Sciences, Ho, Ghana; 4 Department of Health Policy Planning and Management, School of Public Health, University of Ghana, Legon, Ghana; Northeastern University, UNITED STATES

## Abstract

Breast cancer is the number one cause of cancer death in women globally. According to the Global cancer registry, there were 2.3 million new cases of breast cancer diagnosed in 2020 worldwide, accounting for 25% of all cancer cases in women. The data on the cost burden of breast cancer on households is limited in Ghana, it is therefore imperative that it is estimated to ensure effective planning and provision of adequate resources for breast cancer treatment. This cost-of-illness study estimates the household treatment cost of breast cancer and the cost coping strategies used by patients. This cost-of-illness study was conducted at the surgical unit (Surgical unit 2) of the Korle Bu Teaching Hospital (KBTH), with 74 randomly selected patients and their accompanying caregiver(s). Data was collected using structured questionnaire on direct, indirect and intangible costs incurred and coping strategies used by patients and their households. The results are presented in descriptive and analytic cost statistics. Most of the patients were aged 40–69 years and were married with moderate education levels. Nearly 57% of patients earn an income of USD 370 or less per month. The average household expenditure was USD 990.40 (medical cost: USD 789.78; non-medical cost: USD 150.73; and indirect cost: USD 50). The publicly provided mechanism was the most utilized cost coping strategy. The direct, indirect and intangible costs associated with breast cancer treatment had significant financial and psychological implications on patients and their households. Moreover, poorer families are more likely to use the publicly provided strategies to cope with the increasing cost of breast cancer treatment.

## Introduction

Breast cancer is the number one cause of cancer death in women globally [1]. According to the Global cancer registry, there were 2.3 million new breast cancer cases in 2020, accounting for 25% of all cancer cases in women [2]. The incidence of breast cancer is highest in the Australian subcontinent, Northern, Western, Southern Europe and the Americas with South Asia and Latin America being the continents with the highest global burden [3,4]. These numbers were higher than observations seen in North America. Moreover, it was apparent that countries in South Asia recorded the highest welfare losses to breast cancer. The mortality trends of breast cancer had been relatively stable and even decreasing in many developed countries over the past 20 years. However, in developing countries of Africa, Asia, and southern America the incidence of breast cancer has been on the ascendency. The age-adjusted death rate in Africa is the highest worldwide, with Nigeria having the highest death rate [5]. In Ghana, there were 4,482 new breast cancer cases in 2020 according to the Global cancer registry which accounted for 32% of all female cancers. It earned the unenviable reputation as the number one cause of cancer death in Ghanaian women [6].

The economic cost of morbidity and mortality from cancer globally was USD 895 billion in 2008 [7]. This figure accounts for 1.5% of global GDP. The productivity losses resulting from breast cancer remain the largest loss to the global economy [7]. Luengo-Fernandez and colleagues [7], found that the cost burden of lung, breast, colorectal, and prostate cancers in the European Union was about USD 65 billion in 2009, accounting for 44% of the economic cost of cancer in the European Union. Furthermore, Liao and colleagues [8] estimated the overall average expenditure for breast cancer in China to be USD 8,450 (medical expenditure: USD 7527; non-medical expenditure: USD 922); the productivity time lost was estimated at USD 1,529. A similar study in Pakistan [9], estimated the average direct medical care cost to be USD 1,262.18, followed by the average direct non-medical care cost of USD 310.88 and indirect cost of USD 273.88.

Ghana currently has no established national cancer registry; therefore, the total number of breast cancer cases diagnosed annually is unknown. However, Korle Bu Teaching Hospital (KBTH), one of two major referral facilities, treats an average of 400 new breast cancer cases per year at its surgical unit. Few studies have been done to estimate the economic burden of breast cancer in Ghana. Hughes et al [10], in a study found the direct annual cost per patient to be USD 1,084 and the indirect annual cost to be USD 2,061. From the study by Zelle *et al* [11], at the KBTH, it was realized that twice-yearly screening of women aged between 40 and 69 years, in addition to the treatment of all stages, was cost-effective (USD 1,299 per DALY averted). An earlier Ghanaian study also suggested that the delayed presentation of cases is a culmination of several factors: 57% of respondents absconded because of the fear of surgery (mastectomy), another 37% resorted to herbal treatment, and 30% sought solace in prayer camps, reporting back in the advanced stages of the disease which may affect the cost of treatment [12]. Earlier studies focused on the cost of treatment without regard to the containment measures used by patients to cope with the increasing cost. In this study, the cost coping strategies adopted by patients and their families were ascertained in addition to estimating the household treatment cost of the disease.

This study aimed to estimate the household direct, indirect and intangible costs from the patient perspective and the cost coping strategies associated with breast cancer treatment at the Korle Bu Teaching Hospital.

## Materials and methods

### Ethics statement

Ethical approval was obtained from the Korle Bu Teaching Hospital Institutional Review Board with certificate number: KBTH-STC/IRB/00043/2019. A written formal consent was obtained from all study participants.

This was a cross-sectional cost-of-illness study from the patient perspective. A structured cost and coping strategy questionnaire collected data from 76 randomly selected patients through face-to-face interviews. Patients were recruited from May to August, 2019.

#### Study area and setting

The study was conducted at the surgical unit (surgical unit 2) of the Korle Bu Teaching Hospital (KBTH). Established in 1923, KBTH is the foremost teaching hospital and the largest referral center in Ghana. It has a bed capacity of 2000 and daily sees 1500 patients at its outpatient departments. The surgical unit diagnosed and managed 347 new breast cancer cases in 2016. The hospital is located in the capital, Accra.

#### Cost-of-illness analytic framework

Breast cancer patients incur three kinds of costs—direct, indirect and intangible. According to Fautrel *et al*. [13], direct health services costs are primarily medical and non-medical costs. Direct medical costs represent the costs of services such as consultation, diagnosis, cost of prescriptions, palliative care etc. Direct non-medical costs refer to expenditure on food and drinks, lodging, transportation etc. Indirect cost refers to productivity losses, time lost due to travelling, and doctor-waiting time. Productivity losses encompass the individual’s or household members’ loss of working time due to the illness.

**Fig 1:**
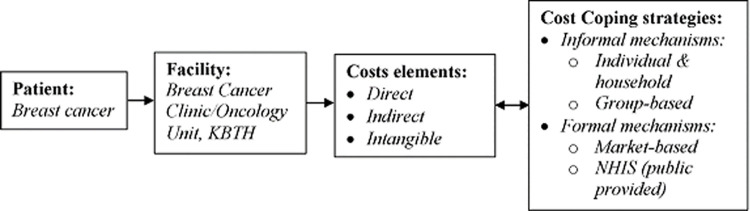
Analytical framework of household cost and coping strategy of breast cancer treatment.

The intangible cost focuses on the psychological impact of the disease, such as stress, fear, depression, physical pain, etc. The second component of this study is the cost coping strategies adopted by patients: This is mainly formal (i.e., market-based and public provided) and informal mechanisms (i.e., individual and household and group-based). The cost component is closely linked with coping strategy to ensure synergy in breast cancer management. The cost-of-illness and coping strategy analytical framework is shown in Fig 1.

#### Sampling

Sample size was based on a previous study by Niens *et al* [14], which looked at treatment outcomes for breast cancer management at the Komfo Anokye Teaching Hospital. Although, the study by Niens *et al* [14] was on treatment outcomes, we deemed it expedient to use their work as a base for our study because of the unavailability of comprehensive data on cost of treatment studies in Ghana relating to breast cancer.

The study was designed to randomly select 76 patients assuming a 10% non-response rate, using a standard deviation of 127.50, at a 95% confidence interval (Z score = 1.96) and a margin of error of 30.

#### Method of data collection

The seventy-six patients (76) were selected by simple random sampling using a random number table. The table was followed chronologically, from top to bottom; the first 76 numbers on the random number table coinciding with specific folder numbers were selected.

#### Method of data collection

The study used a quantitative data collection approach. The structured questionnaire consisted of three sections, namely 1) socio-demographic characteristics, 2) cost information and 3) cost coping strategy mechanisms adopted. The medical cost comprised consultation, laboratory, surgery and others, and the non-medical expenditure was made up of transportation, food, lodging and rent. The indirect costs covered travel and waiting times and productivity days lost from work in seeking treatment. The intangible cost measuring the psychological burden (i.e., fear, stress, depression and physical pain) were determined using a 4-dimension Likert scale to each of the questions. The cost coping strategies assessed patient use of formal and informal mechanisms for treatment. Questionnaires were administered through face-to-face interviews after free and written informed consent had been obtained from the patient after being assured of confidentiality, voluntary withdrawal and data security. Data collated was entered into Excel 2013 and exported to STATA version 15 for analysis. We received ethical approval from the Korle Bu Teaching Hospital Institutional Review Board (KBTH-STC/IRB/00043/2019). Quality control measures instituted prior to data collection were: 1) training of research assistants on questionnaire and consent form administration, 2) pre-testing of questionnaires, and 3) error correction. Completed questionnaires were validated and entered daily. Patients histologically diagnosed as having breast cancer, undergoing treatment, could understand the survey process and consents to participate in the study were included and those who were too ill to communicate or unwilling to participate were excluded. Two incomplete data sets were precluded in the analysis.

#### Data analysis

Table 1 provides a detailed summary of cost and coping strategy analyses undertaken in the study. A sensitivity analysis was conducted as part of the analyses to test the robustness of cost elements. The key cost variables were incrementally varied at 3%, 5% and 7%, as shown in literature [15,16]. Further details are found in Table 1.

**Table 1:** Breast cancer treatment cost and cost coping analyses.

Cost type	Cost component	Cost Estimation Approach
**Direct**	Medical	The medical cost included the costs of consultation, folders, prescriptions, laboratory investigations, surgery, radiotherapy and chemotherapy. These costs were summed up, and to obtain the average cost per patient, it was divided by the total number of respondents.
	Non-medical	The non-medical costs were transportation, lodging/rent, food and drinks. The transportation cost was calculated by summing the travel costs incurred by the patients and accompanying caregiver(s) covering all visits to and from the hospital. The lodging/rent cost was the summation of such costs incurred by the patient and accompanying caregiver(s) during visits to the hospital for treatment. The cost of food and drinks was calculated by summing the costs incurred by the patient and accompanying caregiver(s) on food and drinks during their visits to the hospital for treatment. The total non-medical cost was obtained by summing the cost incurred by patients and accompanying caregiver(s) on travel, lodging/rent, food and drinks due to their visit to the hospital for treatment. The average cost per patient was obtained by dividing the total non-medical cost by the total number of respondents.
**Indirect**	Productivity losses due to health care seeking	Human capital approach was used to estimate the productivity losses by patient and accompanying caregiver(s). This was the sum of hours spent seeking treatment by the patient and accompanying caregiver(s) (travel, waiting and treatment times) during hospital visits. The mean cost was determined by dividing the indirect cost by the number of respondents.
	Other productivity losses	This was sum of total number of other productive work days lost (i.e., absenteeism) to patient and accompanying caregiver(s) for hospital visit.
	Total indirect valuation	The total hours productivity losses (i.e., seeking health care and other productivity losses) was determined by multiplying by the average hourly earnings–minimum wage (i.e., USD1.95 per day).
	Total treatment cost	This was the summation of the total direct and total indirect costs.
**Sensitivity analysis**	One-way and multi-way sensitivity analyses were conducted to test the robustness of the cost estimates. Key cost variables such as the costs of chemotherapy and the daily minimum wage associated with uncertainty and variations in the data collected were used. These variables were incrementally varied from 3%, 5% and 7% as shown in literature [15,16] to ascertain their effect on the total cost calculated.	
**Intangible**	Composite intangible cost	The composite intangible cost was developed from responses to 4-dimension Likert scale questions (i.e. [1], Not at all [2], Mildly [3], Moderately [4], Severely/Extremely) in relation to fear, stress, depression and physical pain. The aggregated score from these four questions were re-categorized into ‘Low’ (12–24), ‘Moderate’ (28–37) and ‘High’ (38–48) cost using the descriptive statistics tertile approach.
**Coping strategies used**	Cost coping strategies are divided into two main groups (i.e., informal and formal mechanisms). The informal mechanisms were further divided into the Individuals & household and Group-based categories and the formal mechanisms, subdivided into Market-based facility and the Publicly provided strategy. The total responses to these items were collated, and proportions of the four categories were calculated.	

## Results

### Background characteristics

Table 2 shows the socio-demographic characteristics of patients. The achieved response rate was 97% (74). About 74% of the patients were aged 40–69 years, and 46% (34) were married. Nearly 45% had obtained JHS/Middle school education, and 48.6% (36) were self-employed. A majority, 82.4% (61), of the patients were Christians. Nearly 57% (42) had an income of USD 370 or less per month, whilst 28% (21) had no source of income. The socio-demographic characteristics were compared to findings from previous studies. The absence of a parameter in a field means it was unavailable.

**Table 2:** Socio-demographic characteristics of breast cancer patients.

Characteristics	Number (N = 74)	Percentage (%)	Dedey et al [17] (N = 205) Percentage (%)	Mburu et al [18] (N = 31) Percentage (%)
Age (years):				
30–39	11	14.9	21.2	
40–49	18	24.3	26.1	
50–59	19	25.7	27.9	
60–69	18	24.3	24.8	
>69	8	10.8		
Mean age ±SD	53.6 ± 11.96		51.1 ± 11.8	51
Marital Status:				
Single	12	16.2		
Married	34	46.0	63.6	71
Divorced	11	14.9		29
Widowed	10	13.5		
Separated	7	9.5		
Educational status:				
No education	9	12.2	18.5	
Primary	8	10.8	6	58
JHS/Middle school	33	44.6	37	10
SHS	5	6.8	19	
Tertiary	19	25.7	19.5	22
Employment status:				
Unemployed	21	28.4	38.8	55
Private Sector Employee	6	8.1		45
Public Sector Employee	11	14.9		
Self Employed	36	48.6		
Religion:				
Muslim	11	14.9	14.7	10
Traditionalist	2	2.7	0.5	
Christian	61	82.4	84.8	90
Income group (USD):				
<USD 370	42	56.8	20.7	
>USD 370	11	14.8	2.3	
Unemployed	21	28	38.8	55
Don’t know			19.1	
Unwilling to disclose			19.1	
NHIS subscription Yes NO	71 3	96 4	88.6 11.4	97 3
Total:	74	100.0	100	100

### Direct cost of breast cancer treatment

Table 3 shows that the direct cost for treating breast cancer patients was approximately USD69, 595. The direct cost comprises the medical cost estimated at USD 58,440 and the non-medical cost estimated at USD 11,154. Therefore, each household is estimated to spend on average USD 940 as direct cost when seeking treatment. Overall, the medical and direct costs make up 84% and 95% of the total expenditure, respectively.

**Table 3:** Direct and indirect cost.

Type of costs	Total (USD)	Mean cost (USD)	Standard deviation	Median cost(USD)	Cost profile (%)
**Direct costs:**					
**Direct medical:**					
Consultation	1,307.33	17.67	45.75	0.0	1.8
Investigations	25,502.75	344.63	171.52	329.67	34.8
Treatment	31,630.22	427.44	449.89	377.29	43.2
Total medical:	58,440.30	789.74	667.16	706.96	79.8
**Direct non-medical:**					
Transportation	5,253.66	70.99	100.86	18.31	7.1
Food and drinks	4,834.62	65.33	83.94	5.04	6.6
Lodging/rent	1,065.93	14.41	107.56	0.0	1.5
Total non-medical:	11,154.21	150.73	213.15	27.47	15.2
Total direct cost:	**69,594.51**	**940.47**	**535.40**	**831.50**	**95.0**
**Indirect cost:**					
Patient workdays lost (per month)	290.64	3.92	11.76	0.0	0.4
Travel	57.42	0.77	0.97	0.49	0.1
Waiting	55.20	0.75	0.31	0.86	0.0
Accompanying persons workdays lost (per month)	3,288.63	44.45	87.00	6.10	4.5
Total indirect:	3,691.89	49.89	86.83	8.90	5.0
**Grand total:**	**73,286.40**	**990.36**	**544.65**	**907.99**	**100**

### Indirect cost of breast cancer treatment

The total productivity losses concerning patients and accompanying relatives is estimated at 2,865.9 hours, an equivalent of 4.8 working days lost per patient and accompanying relatives per month. Therefore, the indirect cost, a cost expression of the productivity losses and time spent seeking treatment, is approximately USD 403.30. The losses attributable to accompanying relatives are also estimated at USD 3,288.60. Consequently, the total indirect cost is estimated at USD 3,691.89 with a mean indirect cost of USD 49.89.These findings are illustrated in Table 3. Overall, all patients and their accompanying relatives incurred a total cost of USD 73,286.40. Hence, the average household expenditure on breast cancer treatment is estimated at USD 990.

### Intangible cost of breast cancer treatment

**Fig 2:**
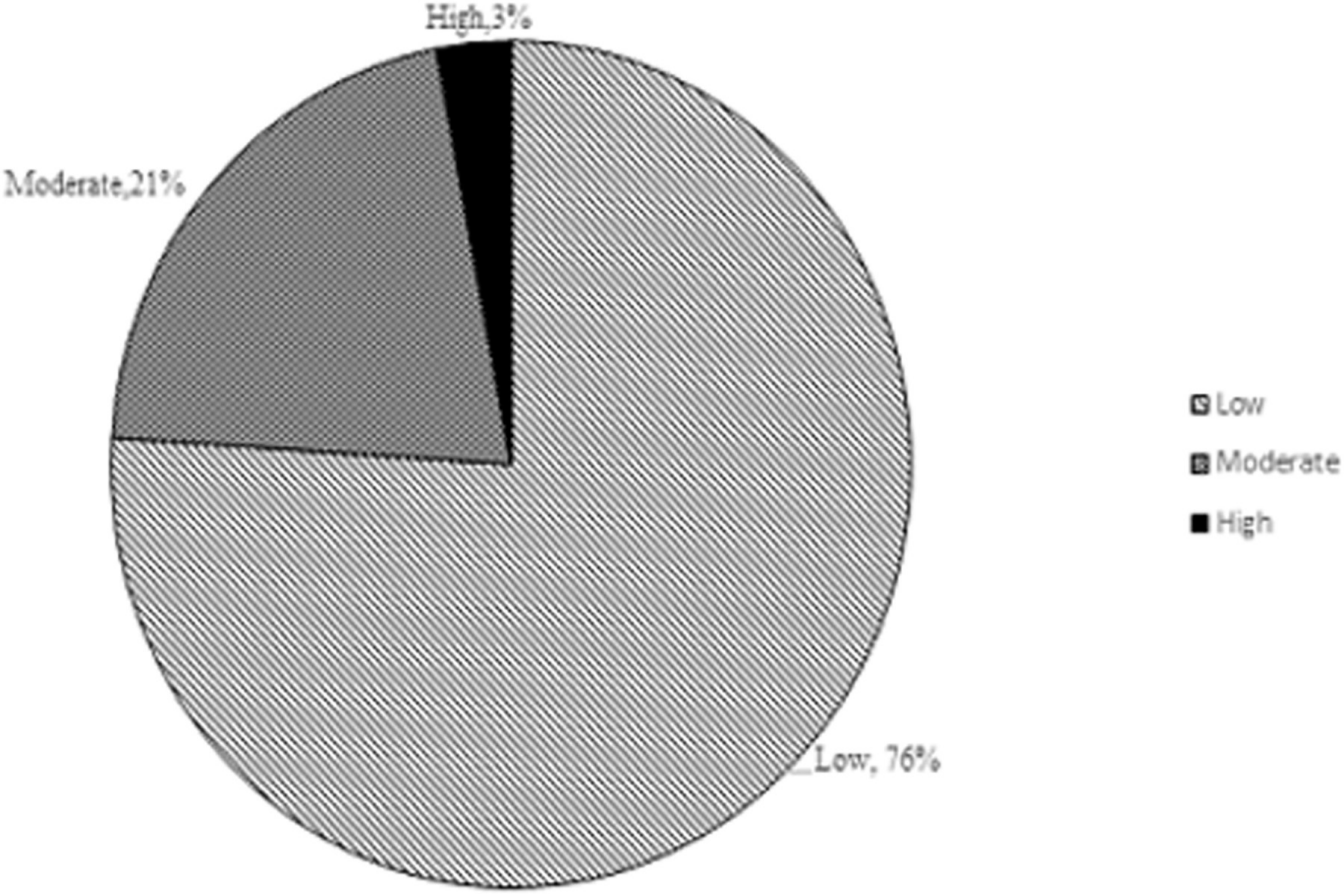
Intangible cost of breast cancer treatments.

Fig 2 shows that 76% of patients reported low intangible cost, 21% moderate cost and 3% experienced a high cost.

### Cost coping strategies of breast cancer patients

**Fig 3:**
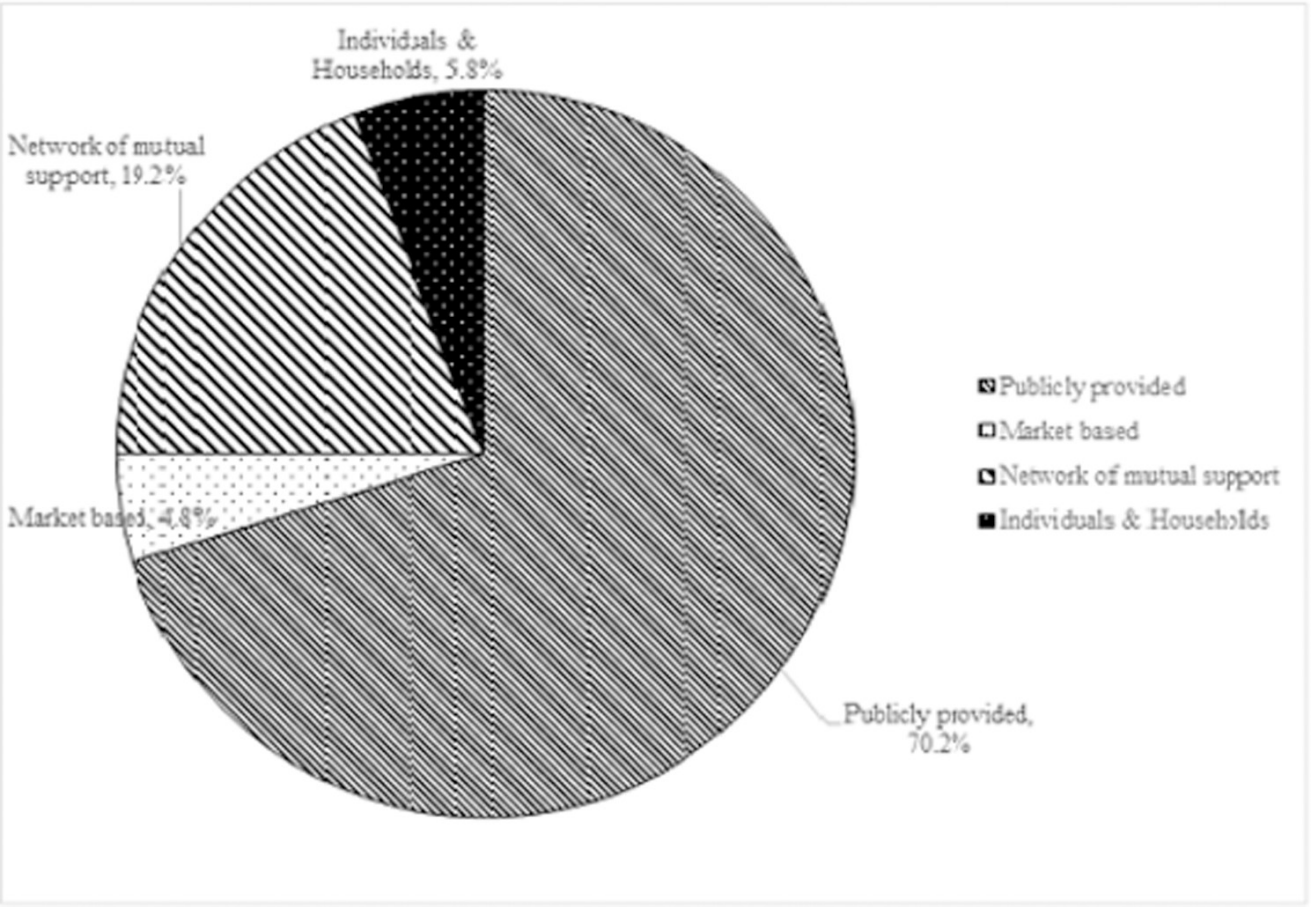
Patient cost coping strategies.

Fig 3 shows patient cost coping strategies used. About 70% used the publicly provided strategy of which, 96% (71) had valid NHIS membership cards. Market-based strategies such as sale of financial assets and loans from financial institutions were used less (4.8%).

## Discussion

The treatment for breast cancer accounted for an average household cost of USD 990.40, direct cost was estimated at USD 940.40 and indirect cost was an average of USD 50.00. Most respondents reported a low intangible cost (76%). From the foregoing, it can be argued that the disease is costly to both households and the state hence the need for strategic interventions to fund breast cancer treatment, so as to reduce the economic burden on struggling families.

The relatively young age of participants (mean 53.6, median 54 years and approximately 65% below the retirement age of 60) is indicative that breast cancer is diagnosed at a younger age in Ghana compared to the developed world. This assertion is confirmed by previous studies [10,12]. The mean age observed in our patients was comparable to data from similar breast cancer patients’ population across Ghana [17,18]. Also consistent with our findings, Dedey et al [17] and Mburu et al [18] reported that majority of breast cancer patients subscribed to the national health insurance scheme (89% & 97% respectively), which is at variance with the estimated national enrollment figure for women (66%) [19]. Amoako et al [20] reported on summarized data from the Kumasi Cancer Registry and found that breast cancer accounted for 30% of all female cancers and was the leading cause of cancer in the Kumasi metropolis of Ghana. They further estimated the incidence rate of breast cancer in the Kumasi metropolis to be 16.1 per 100,000 population. Analysis also show a correlation between income levels and treatment cost. Individuals who earn more than USD 370 are likely to spend more on healthcare (USD 1,158.00) on average, compared to those who earn less (USD 881.00). High income earners spent 1.3 times more than low income earners in the treatment of breast cancer (Tables A and B in S1 Table).

Meanwhile, the cost of surgery constituted about 23% of the total medical cost input, with chemotherapy, prescriptions and imaging making up 6%, 9% and 16% respectively. Patients are afflicted by a double burden, namely the psychological burden of the disfigurement of surgery and the financial implications of it. Be that as it may, they are also confronted by the escalating cost of chemotherapy. In 2016, patients paid an average amount of USD 64.29 for chemotherapy, surgery and radiotherapy [21]. However, the average cost of chemotherapy only, in this study is USD 46.15, which is more than half the combined cost of chemotherapy, surgery and radiotherapy in 2016. Consequently, the cost of treatment borne by patient is increasing yearly even though the economic fundamentals remain basically the same.

The average non-medical cost reported in China [8] is about 11% of the total direct cost compared to 16% in this study. The cost of transportation (47%) forms a significant proportion of the non-medical cost, followed by the expenditure on food and drinks (43%). Together, they constitute 90% of the total non-medical cost, suggesting that food and transportation are essential to breast cancer patients. The average total direct cost comprises 95% of the total cost input. This observation is consistent with findings by Liao [8], Mahmood [9] and Gyau [21].

A multivariate sensitivity analysis on cost of chemotherapy and the minimum wage showed that 3%, 5% & 7% increments in these two elements could results in a 0.3–0.7% increment in the total cost. Looking at the escalating cost of chemotherapy, the sensitivity analysis is all the more meaningful. Patients are likely to bear the brunt of steeper increases in treatment cost unless containment measures are instituted to forestall it (S2 Table).

The total indirect cost constitutes 5% of the total cost input. The total productivity losses were an equivalent of 4.8 working days lost per patient per month. Patients absented themselves for a total of 149 days due to sickness and spent an accumulated time of 236 hours travelling to and from the hospital each month. The average time spent waiting for health personnel is 3 hours per patient. Gyau [21] estimated the total time lost to breast cancer to be 5.6 working days per patient in a similar study. Ekwueme et al [22] showed that work lost per capita is higher in younger employed women than in older women. Breast cancer in young women generally tends to be more aggressive, requiring more elaborate treatment precipitating various physical and psychosocial effects which may culminate in prolonged absenteeism.

Emotional and family support is critical to patient diagnosed with breast cancer, however, estimating the psychological effect is challenging because its value cannot be expressed in monetary terms. Although, all patients reported some level of psychological problem, the average effect is on the level of mild to moderate impact rather than high. This is in line with previous scientific work on psychological effects of breast cancer by Cvetkovic and Nenadovic [23] However, most women adapt well to the diagnosis of breast cancer and manage to endure the complex and sometimes toxic medications associated with initial treatment and recurrence [24]. Nevertheless, a number of women in the present study had to quit their jobs as a result of the disease.

It is also instructive to note that most patients adopted the publicly provided strategy as a cost coping measure (70%). This emanated partly from the observation that the vast majority of patients subscribed to the NHIS (96%, Table 2). Although majority of respondents in this study were of the opinion that the NHIS provided only partial cover for their ailments, a significant number (68 of 74 respondents) were in fair to excellent health (S3 Table). It is inferred that even though the NHIS may not cover the full cost of care as expected by patients it is playing an essential role in improving the health of those enrolled on it. However, in a cost coping study, Mauch and colleagues [25] observed that about 70% of tuberculosis patient in Ghana had to stop work; and resort to borrowing and selling personal assets to cope with cost of treatment. As high as 37% of patients sold property and 50% had to borrow to deal with the escalating cost. Furthermore, Leive & Xu [26] reported that households in African countries with the highest inpatient healthcare spending were at least 10% more likely to borrow and sell assets than those that made no significant out of pocket payments. This effect, they mentioned was most remarkable in Ghana, where households were 40% more likely to borrow or sell property. It must however be noted that these cost coping measures are not mutually exclusive. Patients and households resort to several containment measures to deal with the burden of illness.

Whereas our study is an institutional-based one, limiting the scope of the data collected, KBTH is one of only two major referral facilities for breast cancer treatment; hence, the findings are still relevant. Additionally, the usage of the national minimum wage for determining the indirect cost may have introduced bias in the total cost input since income variations exist across sectors. Finally, due to the lack of comprehensive breast cancer cost studies in Ghana, this study used the cost parameter from Niens et al. [14] to estimate the sample size, which may have influenced the study’s cost estimates. Thus, the generalization of the results must be made cautiously.

## Conclusion

The direct, indirect and intangible costs associated with breast cancer treatment had significant financial and psychological implications on patients and their households. Moreover, poorer families are more likely to use the publicly provided strategies to cope with the increasing cost of breast cancer treatment.

## Supporting information

S1 Table Table A: Income by direct cost (raw data). Table B: Cross-tabulation of income by direct cost.
(DOCX)

S2 Table Sensitivity analysis. (DOCX)

S3 Table Variables against coping strategies. (DOCX)

S1 Text Questionnaire. (DOCX)
