# Supplementary material for: Household treatment cost of breast cancer and cost coping strategies from a tertiary facility in Ghana
Source: PLOS Glob Public Health. 2022 Mar 9;2(3):e0000268. doi: 10.1371/journal.pgph.0000268 (PMC10022245; doi:10.1371/journal.pgph.0000268)
Supplement: S1 Table — Table A: Income by direct cost (raw data). Table B: Cross-tabulation of income by direct cost. (DOCX) [file pgph.0000268.s001.docx]

**Supporting information**

Table A. Income by Direct cost (Raw data)

| Income brackets | Income (GHS) | Mean | Std Dev. freq. | |
| --- | --- | --- | --- | --- |
|  |  |  |  |  |
|  | <500 | 4728.376 | 2526.039 | 27 |
|  | 500 -1999 | 5011.168 | 1718.247 | 15 |
|  | 2000-4999 | 6436.909 | 2406.587 | 10 |
|  | 5000-10000 | 5166.594 | 0 | 1 |
|  | 0(no income) | 4770.566 | 2959.607 | 21 |
|  |  |  |  |  |
|  | Total | 5034.476 | 2505.426 | 74 |

Table B: Cross-tabulation of income by direct cost

| Income | Direct cost | freq |
| --- | --- | --- |
| < USD 370 | USD 880.91 | 63 |
| >USD370 | USD 1,157.77 | 11 |

1USD : GHS 5.46 June, 2019

USD 370 = GHS 2,000
