# Supplementary material for: Household treatment cost of breast cancer and cost coping strategies from a tertiary facility in Ghana
Source: PLOS Glob Public Health. 2022 Mar 9;2(3):e0000268. doi: 10.1371/journal.pgph.0000268 (PMC10022245; doi:10.1371/journal.pgph.0000268)
Supplement: S2 Table — (DOCX) [file pgph.0000268.s002.docx]

**Supporting information**

|  |  | **Change in parameter** |  | **% change in total cost** | **% of total cost** | | **% change in total cost** | |
| --- | --- | --- | --- | --- | --- | --- | --- | --- |
| **Scenario** | **Cost component** |  | **Total cost** |  | **Direct** | **Indirect** | **Direct** | **Indirect** |
| **Base scenario** | **Total cost** | **0%** | **400143.7** | **0** | **95.0** | **5.0** | **0** | **0** |
| **Variation (One way sensitivity analysis)*** | **Chemotherapy** | 3% | 400705.2 | 0.1 | 95.1 | 4.9 | 0.1 | -0.1 |
|  |  | 5% | 401079.5 | 0.2 | 95.2 | 4.8 | 0.2 | -0.2 |
|  |  | 7% | 401453.8 | 0.3 | 95.3 | 4.7 | 0.3 | -0.3 |
|  |  |  |  |  |  |  |  |  |
|  |  |  |  |  |  |  |  |  |
| **Variation(one way sensitivity analysis)*** | **Wage** | 3% | 400748.4 | 0.2 | 94.8 | 5.2 | -0.2 | 0.2 |
|  |  | 5% | 401151.5 | 0.3 | 94.7 | 5.3 | -0.3 | 0.3 |
|  |  | 7% | 401554.7 | 0.4 | 94.6 | 5.4 | -0.4 | 0.4 |
|  |  |  |  |  |  |  |  |  |
| **Multivariation( Multiway sensitivity analysis)*** | **Chemotherapy & wage** | 3% | 401309.8 | 0.3 | 94.8 | 5.2 | -0.2 | 0.2 |
|  |  | 5% | 402087.3 | 0.5 | 94.7 | 5.3 | -0.3 | 0.3 |
|  |  | 7% | 402864.7 | 0.7 | 94.6 | 5.4 | -0.4 | 0.4 |

S2 Table: sensitivity analysis
