# Supplementary material for: Household treatment cost of breast cancer and cost coping strategies from a tertiary facility in Ghana
Source: PLOS Glob Public Health. 2022 Mar 9;2(3):e0000268. doi: 10.1371/journal.pgph.0000268 (PMC10022245; doi:10.1371/journal.pgph.0000268)
Supplement: S3 Table — (DOCX) [file pgph.0000268.s003.docx]

**Supporting information**

S3 Table: variables against cost coping strategies

| Items | Coping strategies | | | |
| --- | --- | --- | --- | --- |
|  | Publicly provided | Market based | Network of mutual support | Individuals & Households |
| Age (years) |  |  |  |  |
| <12 |  |  |  |  |
| >12 | 74 | 5 | 20 | 6 |
| Marital status |  |  |  |  |
| Married | 35 | 2 | 11 | 3 |
| Not married | 39 | 3 | 9 | 3 |
| Income (USD) |  |  |  |  |
| < 370 | 43 | 5 | 11 | 4 |
| >370 | 11 | 0 | 3 | 1 |
| No income | 20 | 0 | 6 | 1 |
| Reported physical health |  |  |  |  |
| Poor | 6 | 0 | 1 | 0 |
| fair | 29 | 2 | 6 | 3 |
| good | 37 | 3 | 12 | 3 |
| Excellent | 2 | 0 | 1 | 0 |
| Reported living situation |  |  |  |  |
| Comfortable | 32 | 1 | 11 | 1 |
| poor | 42 | 4 | 9 | 5 |
