# Supplementary material for: Household treatment cost of breast cancer and cost coping strategies from a tertiary facility in Ghana
Source: PLOS Glob Public Health. 2022 Mar 9;2(3):e0000268. doi: 10.1371/journal.pgph.0000268 (PMC10022245; doi:10.1371/journal.pgph.0000268)
Supplement: S1 Text — (DOCX) [file pgph.0000268.s004.docx]

**QUESTIONNAIRE**

**TITLE: Household Treatment cost of Breast Cancer and Cost Coping Strategy of Women, presenting at the Korle Bu Teaching Hospital.**

I am Kekeli Adanu, a student of the School of Public Health, conducting a research on the treatment cost of Breast cancer and the Cost Coping Strategies adopted by patient and their household when undergoing treatment for this condition.

I wish you could spare sometime and participate in this study. You are assured of the highest level of privacy and confidentiality.

Thank you

Respondent Code …………………………….. Date of Interview (dd/mm/yyyy) ……/……/……….

**SECTION A: SOCIO-DEMOGRAPHY STATUS**

| No | Questions | Responses |
| --- | --- | --- |
| 1 | Name (Initials) |  |
| 2 | Age as at last birthday : | …………… |
| 3. | Sex   1. Male 2. Female | [ ] |
| 4 | Marital Status   1. Single 2. Married 3. Divorced   4. Widowed  5. Separated | [ ] |
| 5 | Educational Status   1. No Education 2. Primary   3. JHS/Middle School  4. SHS  5. Tertiary | [ ] |
| 6 | Religion   1. Muslim 2. Traditionalist 3. Christian 4. Other | [ ] |
| 7 | Employment Status   1. Unemployed 2. Private Sector Employee 3. Public Sector Employee 4. Self-Employed   If unemployed, skip to Q 11 | [ ] |
| 8 | If Employed, what is your occupation?   1. Health 2. Civil Service 3. Security services   4. Business/ Petty trading  5. Banking  6. Other, Please Specify…………………………………….. | [ ] |
| 9 | If employed, are you still working despite your illness?   1. Yes 2. No | [ ] |
| 10 | How much do you earn monthly?   1. Less than GHS 500 2. GHS 500 – GHS 1999   3. GHS 2000 – GHS 4999  4. GHS 5000 – GHS 10,000  5. > GHS 10,000 | [ ] |
| 11 | Do you have a valid NHIS card?   1. Yes 2. No | [ ] |
| 12 | When were you diagnosed as having breast cancer? | …………. years |
| 13 | Is there a family history of breast cancer?   1. Yes 2. No   If No, skip to Q 15 | [ ] |
| 14 | If yes to 13 above, please specify ……………………………………. |  |
| 15 | For how long have you been on treatment? | ………..yrs ……….mnths |
| 16 | What is the source of funding for your treatment? (tick as many as applicable)   1. Self [ ] 1. NHIS [ ] 1. Pension [ ] 1. Remittances [ ] 2. Family Support [ ] 1. Private Insurance [ ]   1. Donations/Gifts [ ] |  |
| 17 | How would you classify your household’s financial situation these days?  [1] Very comfortable  [2] Comfortable  [3] Poor  [4] Extremely poor | [ ] |
| 18 | How do you rate your own physical health now?  [1] Poor [2] fair [3] good [4] excellent | [ ] |

**SECTION B - DIRECT COST**

MEDICAL

19. How much did you and your household spend on the following in a month?

| No | Item | Cost |
| --- | --- | --- |
| a | Folder |  |
| b | Consultation |  |
| c | Prescriptions per month |  |
| d | Laboratory Investigations per month |  |
| e | Imaging studies |  |
| f | Histopathology |  |
| g | Chemotherapy per month |  |
| h | Surgery (mastectomy/breast conservation surgery) |  |
| i | Radiotherapy per month |  |
| j | Others (please specify) |  |

**NON –MEDICAL**

20. How much was spent on the following?

| No | Item | Cost |
| --- | --- | --- |
| a | Transportation |  |
| b | Food and drinks |  |
| c | Lodging/rent |  |
| d | Others (specify) |  |

**SECTION C – INDIRECT COST**

| No | Item |  |
| --- | --- | --- |
| 21 | How many days did you absent yourself from work within the past month because of your sickness? | …………… days |
| 22 | On the average, how many days do you absent yourself from work per month on account of your sickness? | ………………days |
| 23 | On the average, how many hours do you spend travelling to and from the hospital each month? | ………………. Hours |
| 24 | On the average, how long do you have to wait before seeing a doctor? | ………….hr(s) ………. mins |

**Person Accompanying Patient**

| 25 | How many people accompany patient to the hospital on each visit?  If None, skip to Q 29 | ……………….. |
| --- | --- | --- |
| 26 | How many days did you absent yourself from work within the past month because of patient’s illness? | ………………. days |
| 27 | On the average, how many hours do you spend travelling together with patient to and from the hospital each month? | ………………. Hours |
| 28 | On the average, how many hours in a day does a household member spend taking care of patient? | ……………….hours |

**SECTION D – INTANGIBLE COST**

29. Pain

| 29 a | I feel pain in my breast   1. Not at all 2. Mildly 3. Moderately 4. Severely | [ ] |
| --- | --- | --- |
| 29 b | I feel pain around my arms   1. Not at all 2. Mildly 3. Moderately 4. Severely | [ ] |
| 29 c | I feel general bodily aches and pains   1. Not at all 2. Mildly 3. Moderately 4. Severely | [ ] |

30. Fear

| 30 a | I am worried about my future   1. Not at all 2. Mildly 3. Moderately 4. Severely | [ ] |
| --- | --- | --- |
| 30 b | I am afraid of dying as a result of my sickness   1. Not at all 2. A little 3. Moderately 4 Extremely | [ ] |
| 30 c | I worry that the cancer may spread to other part of my body   1. Not at all 2. Mildly 3. Moderately 4. Extremely | [ ] |
| 30 d | I am worried that my children may inherit the condition   1. Not at all 2. Mildly 3. Moderately 4. Extremely | [ ] |

31. Depression

| 31 a. | I feel is not worth living anymore   1. Not at all 2. Mildly 3. Moderately 4. Severely | [ ] |
| --- | --- | --- |
| 31 b. | I feel I am a burden onto those who take care of me   1. Not at all 2. Mildly 3. Moderately 4. Severely | [ ] |
| 31 c. | I feel my partner does not find me attractive any more   1. Not at all 2. Mildly 3. Moderately 4. Extremely | [ ] |
| 31 d. | I sometimes feel like committing suicide   1. Not at all 2. Mildly 3. Moderately 4. Severely | [ ] |

32. Stress

| 32 a. | I feel stressed out by my condition   1. Not at all 2. Mildly 3. Moderately 4. Severely | [ ] |
| --- | --- | --- |

**Cost Coping Strategies**

33. Individual and Household

| 33 a | Did you or any household member have to sell a property to cater for your illness?   1. Yes 2. No   If No, skip to Q33c | [ ] |
| --- | --- | --- |
| 33 b | If Yes above, please specify item and how much was realized  ……………………………………………………………………………………………. |  |
| 33 c | Did you or any household member have to take money from lenders?   1. Yes 2. No   If No, skip to Q 34 | [ ] |
| 33 d | If Yes above, please specify amount  ……………………………………………………………………………………. |  |
|  |  |  |
| 34 | Market Based |  |
| 34 a | Did you have to take loans from any financial institution to take care of your illness?   1. Yes 2. No   If No, please skip to Q34 c | [ ] |
| 34 b | If Yes to above, how much …………………………………. |  |
| 34 c | Did you have to sell any financial assets to take care of your illness?   1. Yes 2. No   If No, please skip to Q 34e | [ ] |
| 34 d | If Yes, please specify  ………………………………………………………………………………….. |  |
|  |  |  |
| 35 | Networks of Mutual Support |  |
| 35 a | Are you a member of any group/Club   1. Yes 2. No   If No, skip to Q 35 d | [ ] |
| 35 b | If Yes to ‘g’ above, have you receive any financial support from this group to take care of your illness?   1. Yes 2. No | [ ] |
| 35 c | If Yes, How much were you given?......................................... |  |
| 35 d | Have you received any support from any religious grouping (eg church/mosque)?   1. Yes 2. No   If No, skip to Q 36 | [ ] |
| 35 e | If Yes, specify amount…………………………………………………… |  |
|  |  |  |
| 36 | Publicly Provided |  |
| 36 a | Are you a registered member of the National Health Insurance scheme (NHIS)?   1. Yes 2. No   If No, please skip to Q 36 c | [ ] |
| 36 b | If Yes above, how beneficial has the NHIS been towards treatment of your illness?   1. Not beneficial at all 2. Partially 3. Covers full cost of treatment | [ ] |
| 36 c | Have you received any other form of social support from Government?   1. Yes 2. No   If No, please skip to Q 36 e | [ ] |
| 36 d | If Yes, please specify  …………………………………………………………………………………………. |  |
| 36 e | Have you received any form of support from your workplace?   1. Yes 2. No | [ ] |
| 36 f | If Yes, please specify  ………………………………………………………………………………………………. |  |

Thank You
