# Supplementary material for: Household treatment cost of breast cancer and cost coping strategies from a tertiary facility in Ghana
Source: PLOS Glob Public Health. 2022 Mar 9;2(3):e0000268. doi: 10.1371/journal.pgph.0000268 (PMC10022245; doi:10.1371/journal.pgph.0000268)
Supplement: Attachment — Submitted filename: Response to reviewers plosgbh.docx [file pgph.0000268.s005.docx]

**UNIVERSITY OF HEALTH AND ALLIED SCIENCES**

**SCHOOL OF MEDICINE**

**Department of Surgery**

**PMB 31**

**Ho, Volta Region**

**Ghana**

**Tel: 233506816784**

**Email: kkadanu@uhas.edu.gh**

5^th^ February, 2022

The Editor

PLOS Global Public Health

Dear Sir/Madam,

**RESPONSE TO REVIEWERS COMMENT**

I write to respond to comments made by the editor and reviewer(s) to our manuscript titled “Household Treatment Cost of Breast cancer and Cost Coping Strategies from a Tertiary facility in Ghana”. The line numbers indicated are those found in the revised manuscript marked “revised manuscript with track changes”. In addition, we have revised the authors list to reflect the contribution of Prof. Moses Aikins to this work.

| Comments | Response |
| --- | --- |
| Editor’s comments | |
| 1. Please indicate the time period for enrollment/recruitment of patients into the study | Time period provided. Please see line number 105 |
| 2. - It would be useful to compare the characteristics of patients in the study to breast cancer patients more broadly in Accra/Ghana. In Table 2, please add a comparison patient population (either from all of Accra or Ghana over a single comparable year for example), and please comment briefly in the Discussion section on the similarities/differences in the characteristics. | Two comparative populations provided in Table 2, from publications by Dedey et al (2016) and Mburu et al (2021). Brief description of similarities/differences to our data provided in the discussion. Please see line numbers 281 – 285. Our attempts to obtain data from the Accra and Kumasi cancer registries were unsuccessful. We got the impression that the two institutions were currently, not fully operational. We however reported findings by Amoako et al (2019) which summarized data from the Kumasi cancer registry for the year 2015. See line numbers 285 - 289 |
| Reviewers’ comments | |
| 3. From the cancer observatory data, breast cancer is the leading female cancer in Ghana, so please use the current data to incorporate this in your introduction. Kindly highlight the cost coping strategies common in Ghana in terms of healthcare cost. This can include areas not directly related to cancer so that readers can have more insight into the situation in Ghana. Overall the manuscript is well written. I commend the authors for this research. | The introduction has been updated to reflect the role of breast cancer as the leading female cancer in Ghana. Please see line numbers 68 – 71. The literature on global incidence of breast cancer has also been updated. In addition, the discussion has been updated to include the common cost coping strategies adopted by Ghanaians in response to healthcare cost. Please see line numbers 338 - 347 |
| Journals’ requirements | |
| 4. . Please include additional information regarding the survey or questionnaire used in the study and ensure that you have provided sufficient details that others could replicate the analyses. For instance, if you developed a questionnaire as part of this study and it is not under a copyright more restrictive than CC-BY, please include a copy, in both the original language and English, as Supporting Information | The questionnaire used for data collection provided as a supporting file |
| 5. Please provide the full name of the IRB. | The full name of the IRB provided, please see line number 155 & 396 |
| 6. The resolution of figures 1-3 is/are very low and somewhat difficult to read. It is important that our Editors and Peer Reviewers are able to read all parts of a submission. Please replace these figures with higher resolution copies. | High resolution replacement for figures 1 – 3 provided using the PACE digital diagnostic tool. |
| 7. Please note that your Data Availability Statement is currently missing the repository name. If your manuscript is accepted for publication, you will be asked to provide these details on a very short timeline. We therefore suggest that you provide this information now, though we will not hold up the peer review process if you are unable. | The repository name ‘Figshare’ has been provided. Please see line number 398 |
| 8. Please review your reference list to ensure that it is complete and correct. If you have cited papers that have been retracted, please include the rationale for doing so in the manuscript text, or remove these references and replace them with relevant current references. Any changes to the reference list should be mentioned in the rebuttal letter that accompanies your revised manuscript. If you need to cite a retracted article, indicate the article’s retracted status in the References list and also include a citation and full reference for the retraction notice. | The reference list has been updated. The citation ‘Lyman et al’ was removed and replaced with ‘Fautrel et al’. The Reference No. 4 (Ranganathan et al) has been reviewed to reflect its appropriate citation in literature. In addition, eight new references were added (Dedey et al, Mburu et al, Ayanore et al, Leive & Xu, Mauch et al, Amoako et al and two references from Global cancer observatory- reference 2& 6).There is no retracted paper in the current reference list. |

Yours sincerely

Dr. Kekeli Kodjo Adanu

Corresponding Author
